# Supplementary material for: Development of chemokine network inhibitors using combinatorial saturation mutagenesis
Source: Commun Biol. 2025 Apr 3;8:549. doi: 10.1038/s42003-025-07778-6 (PMC11969024; doi:10.1038/s42003-025-07778-6)
Supplement: Supplementary file 4 — Supplementary Data 2 [file 42003_2025_7778_MOESM4_ESM.zip › SupplementaryData3/scripts/xattune_ic50.tar!/./script/analysis/JK/JK_R30_E67_INS_HD2_THP1_plot.pdf]

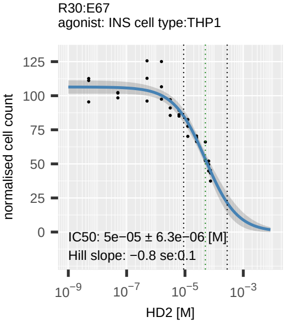
R30:E67
agonist: INS cell type:THP1
normalised cell count
125
100
75
50
25
0
IC50: 5e−05 ± 6.3e−06 [M]
Hill slope: −0.8 se:0.1
10−9
10−7
10−5
10−3
HD2 [M]
